# RNA Editing and Its Roles in Plant Organelles

**DOI:** 10.3389/fgene.2021.757109

**Published:** 2021-09-29

**Authors:** Wei Hao, Guoxiang Liu, Weipeng Wang, Wei Shen, Yuping Zhao, Jialiang Sun, Qiuyue Yang, Yaxin Zhang, Wenjia Fan, Shuaishuai Pei, Zhuanqing Chen, Dongbei Xu, Tengfei Qin

**Affiliations:** ^1^College of Medical Technology, Beihua University, Jilin City, China; ^2^Key Laboratory of Tobacco Improvement and Biotechnology, Tobacco Research Institute of Chinese Academy of Agricultural Sciences, Qingdao, China; ^3^Henan Collaborative Innovation Center of Modern Biological Breeding, Henan Institute of Sciences and Technology, Xinxiang, China; ^4^State Key Laboratory of Agrobiotechnology, School of Life Sciences, The Chinese University of Hong Kong, Hong Kong, SAR China; ^5^Beijing City River and Lake Management Office, Beijing, China; ^6^College of Agronomy, Sichuan Agricultural University, Chengdu, China

**Keywords:** RNA editing, factors, mechanism, plant organelles, deep sequencing

## Abstract

RNA editing, a vital supplement to the central dogma, yields genetic information on RNA products that are different from their DNA templates. The conversion of C-to-U in mitochondria and plastids is the main kind of RNA editing in plants. Various factors have been demonstrated to be involved in RNA editing. In this minireview, we summarized the factors and mechanisms involved in RNA editing in plant organelles. Recently, the rapid development of deep sequencing has revealed many RNA editing events in plant organelles, and we further reviewed these events identified through deep sequencing data. Numerous studies have shown that RNA editing plays essential roles in diverse processes, such as the biogenesis of chloroplasts and mitochondria, seed development, and stress and hormone responses. Finally, we discussed the functions of RNA editing in plant organelles.

## Introduction

Transcribed RNAs need to undergo a series of processes, such as modification, splicing, and editing, to form mature RNAs. RNA editing, a phenomenon that changes genetic information through nucleotide insertion, deletion, or conversion in messenger RNAs of functional genes, is an important supplement to the central dogma (Keller et al., 1999). In 1986, Benne et al. reported that extra nucleotides were added to the frameshift *coxII* gene through the RNA editing process in trypanosome mitochondria (Benne et al., 1986). To date, different types of RNA editing have been widely discovered in divergent RNAs from diverse organisms, including humans, mice, zebrafish, and plants (Yan et al., 2018; Popitsch et al., 2020).

RNA editing can occur in different forms, including the conversion of cytidine (C) to uridine (U), U-to-C, and adenosine (A) to inosine (I), as well as insertions and deletions of U and insertions of guanosine (G; Small et al., 2020). C-to-U editing events have been largely reported in humans. This editing of apolipoprotein-B (apo-B) mRNA was found to be catalyzed by an enzyme complex. The complex contains the catalytic subunit, deaminase APOBEC1, and the subunit apobec-1 complementation factor for RNA binding (Mehta and Driscoll, 2002). A-to-I editing was found to be mediated by adenosine deaminase acting on RNA enzymes. Adenosine deaminase acting on RNA deaminases usually contain no more than three catalytic double-stranded RNA binding domains localized to the N-terminus and a catalytic domain localized to the C-terminus. This type of editing occurred in protein-coding regions of a few genes, causing the recoding and subsequent functional alterations of those genes. Common A-to-I RNA editing targets elements within 5′ and 3′UTRs and introns (Nishikura, 2010). U-to-C RNA editing has also been found in several lycophytes and ferns (Knie et al., 2016). Moreover, insertions and deletions of U and insertions of G were reported to exist in protozoa and some viruses, respectively (Small et al., 2020).

Furthermore, RNA editing has been observed in the cell nucleus and cytosol, mitochondria, and plastids. RNA editing mainly occurs in the mitochondria and plastids in plants. The conversion of C-to-U is the main form of RNA editing in plants. This editing phenomenon was described in 1989 in wheat mitochondria (Covello and Gray, 1989). Two years later, Hoch et al. presented evidence that C-to-U editing was responsible for the conversion from a normal ACG codon to an AUG initiation codon in the mRNA of the maize plastome gene *rpl2*. They demonstrated that RNA editing could also occur within plastids. RNA editing phenomena have been widely reported in many plants. RNA editing events have already been observed in *Arabidopsis*, rice, wheat, tobacco, maize, and soybean (Wang et al., 2016; Rodrigues et al., 2020).

Many studies have shown that abnormal RNA editing results in impaired organelle biogenesis, decreased embryo and endosperm development, retarded plant growth, and poor adaptability to abiotic stresses (Yan et al., 2018). These findings indicate that RNA editing plays a vital role in the growth and development of plants. The process of RNA editing between mammals and plant organelles has many similarities. However, plant organelle RNA editing has certain characteristics and machinery. In this review, we briefly summarized the factors involved in plant organelle RNA editing and reviewed the mechanism of RNA editing in plant organelles. In addition, we reviewed RNA editing events identified in plant organelles through deep sequencing data and discussed the functions of RNA editing in plant organelles.

## Factors Involved in Plant Organelle RNA Editing

Many RNA editing factors have been found in plant organelles. The identification of different types of RNA editing factors broadens our understanding of the RNA editing complex.

Pentatricopeptide repeat (PPR) family proteins are site-specific editing factors that recognize and directly bind to the *cis*-element of the target sequences of RNA (Yan et al., 2018). In 2005, the first RNA editing factor in plant organelles, the PPR family protein CRR4, was reported (Kotera et al., 2005). Pentatricopeptide repeat proteins are nucleus encoded but function in either plastids or mitochondria. They have been discovered to have important effects on RNA editing, splicing, and cleavage (Barkan and Small, 2014). Based on the highly conserved domain modules that are essential for deamination of edited cytidine residues at the C-terminus, these proteins can be separated into extended (E) and DYW subtypes (Cheng et al., 2016). The last C-terminal motif of the PPR family proteins binds to specific nucleotides upstream of the editing site in plant organellar genomes.

Multiple organellar RNA editing factor (MORF) was the second RNA editing factor discovered in the mitochondria of angiosperms (Bentolila et al., 2012). MORF was also identified as an RNA editing factor interacting protein. These factors comprise a central conserved domain that is formed by a core of six β-sheets (Yan et al., 2017). However, the roles of MORF members diverged significantly, and mutation of individual MORFs led to distinct RNA editing deficiencies (Takenaka et al., 2012). Several MORFs were found to interact with PPR family proteins, implying that specific complexes are constructed at different RNA editing sites (Glass et al., 2015).

Organelle RNA recognition motif-containing (ORRM) proteins have been revealed to be critical for RNA editing in the organelles of angiosperms. Disruption of ORRM members can severely affect plant growth (Shi et al., 2017). There is an RNA recognition motif in ORRM proteins that enables them to bind to RNA (Hackett et al., 2017). In addition, additional domains of some ORRM members indicate unique roles for the individual proteins. Organelle RNA recognition motif proteins can associate with other RNA editing factors (e.g., ORRM3/4 with MORF8), suggesting the complexity of the plant RNA editing machinery (Shi et al., 2016b).

Protoporphyrinogen IX oxidase 1 (PPO1) catalyzes the conversion of protoporphyrinogen IX into protoporphyrin IX and has also been identified to function in the RNA editing process in plant plastids. A *ppo1* mutant showed defects in 18 plastid RNA targets, leading to the decreased accumulation of the NDH complex and decreased synthesis of chlorophyll (Zhang et al., 2014). PPO1 can interact with MORF proteins but was not found to interact with PPR factors in chloroplasts. Therefore, PPO1 was speculated to have effects on RNA editing efficiency by coordinating the association of MORF proteins in chloroplasts.

Organelle zinc finger 1 (OZ1) is a RanBP2-type zinc finger protein family member. It was observed to act as an important RNA editing factor by interacting with ORRM1 (Sun et al., 2015). The *oz1* mutant showed decreased editing efficiency at 16 sites and impairment at 14 sites in plastids and consequently exhibited a yellow phenotype, suggesting its vital role in plant development. Organelle zinc finger 1 can interact with other factors, including PPR, ORRM1, and MORF proteins, indicating, that is, has a function in the assembly of the plastid RNA editing complex (Sandoval et al., 2019).

NUWA is identified as a P-class PPR protein. NUWA can interact with SLO2 or CLB19 (Guillaumot et al., 2017). The *nuwa* mutants showed a decreased level of RNA editing at various sites in both the plastids and mitochondria of mature leaves. In addition to PPR proteins, NUWA proteins were found to be coimmunoprecipitated with MORF proteins *in vivo*, which revealed their collaboration with other factors in RNA editing (Bayer-Csaszar et al., 2017).

## The Mechanism of Plant Organelle RNA Editing

In plastids of tobacco, editing an ACG codon of the *psbL* mRNA to an AUG codon caused translational initiation. A chimaeric RNA containing *psbL* deletion derivatives and kanamycin resistance genes was constructed to investigate RNA editing in transgenic plants. This plastid transformation technique revealed that 22 nucleotides are sufficient to direct the editing of *psbL* in the chloroplasts of tobacco. Using this approach, *cis*-elements were determined to be vital for RNA editing site recognition. Another *in vitro* study using extracts of tobacco chloroplasts also detected *cis*-elements that are essential for RNA editing (Shikanai, 2006). Furthermore, the *cis*-elements essential for RNA editing in plant mitochondria were also demonstrated. Using an *in vivo* technique, 16 nucleotides upstream and 6 nucleotides downstream were found to be required for the efficient editing of *coxII* mRNA (Farre et al., 2001). Another group of scientists also identified the *cis*-elements that were required for RNA editing using pea mitochondrial extract (Takenaka et al., 2004).

PPR factors function as site recognition factors in plant organelles. Moreover, PPR proteins could also bind to *cis*-elements specifically. Yin et al. identified the crystal structures of PPR10 in two different RNA states. They showed that PPR10 interacts with RNA in the 5′ to 3′ direction for the target single-stranded RNA (ssRNA; Yin et al., 2013). The PPR–RNA complex, together with other factors, such as ORRM proteins and MORF proteins, forms a higher ordered editosome. Multiple organellar RNA editing factor proteins can act as connectors to form heterodimers and homodimers and selectively interact with different PPR proteins. In *Arabidopsis* and maize, evidence has shown that ORRM proteins are involved in RNA editing. ORRM1 contains two truncated MORF domains and one RRM domain. *Arabidopsis* mutants (*orrm1*) and maize mutants (Zm-*orrm1*) lost 12 and 9 RNA editing sites, respectively (Sun et al., 2013). Three ORRM proteins, ORRM2, ORRM3, and ORRM4, were also found to be essential for RNA editing sites in mitochondria (Shi et al., 2016a).

## RNA Editing Events Identified in Plant Organelles From Transcriptome Data

In recent years, with the development of deep sequencing technologies, various plant organellar genomes have been released, resulting in the surprising discovery of a number of novel RNA editing events.

Grimes et al. screened RNA C-to-U editing sites of the tobacco mitochondrial transcriptome by deep sequencing. In total, they identified 635 editing sites, of which 557 were found within protein-coding genes, 73 in noncoding regions, and five in tRNA genes (Grimes et al., 2014). Zheng et al. identified nearly 570 C-to-U editing site mitochondria-encoded ORFs in rice by Sanger sequencing and publicly available RNA-seq data. Among these identified editing sites, 85.41% were identified on one of the first two bases of a codon, thus altering the corresponding amino acid (Zheng et al., 2020). Edera et al. identified 10,217 editing sites within protein-coding genes in the mitochondria by analyzing publicly available RNA-seq data of 17 diverse angiosperms. They revealed that the majority of the RNA editing sites was conserved across these angiosperms except for some specific sites in different species (Edera et al., 2018).

Oldenkott et al. detected more than 3000 C-to-U RNA editing events by analyzing the transcriptome of the chloroplast of the lycophyte *Selaginella uncinata* (Oldenkott et al., 2014). Lin et al. detected 41 C-to-U editing sites within the transcripts of chloroplast genes of *Vigna radiata* using RNA-seq reads, 5 and 34 of which altered one of the first two nucleotides of a codon (Lin et al., 2015a). Chen et al. identified 137 editing sites by screening the whole plastid transcriptomes of moth orchids, among which 93 were novel edits and 79 were on protein-coding genes (Chen et al., 2017).

Using deep sequencing data, Zheng et al. explored the impact of temperature on the RNA editing process in grape organelles. They identified 627 and 122 RNA editing sites in mitochondria and chloroplasts, respectively. They found that the overall editing level and the expression level of most *PPR* genes were negatively correlated with temperature, suggesting decreased RNA editing efficiency at high temperatures. The authors demonstrated that RNA editing events were susceptible to environmental heat stress (Zhang et al., 2020).

## Functions of Plant Organelle RNA Editing

RNA editing events mainly occur at one of the first two positions of the codon, thus affecting the amino acids encoded in plant organelles. By using publicly available RNA-seq data, Edera et al. found that many RNA editing events caused altered amino acids across angiosperm evolution, and this mainly occurred by substituting editing sites with thymidines (Edera et al., 2018).

Some studies have revealed that mitochondrial RNA editing plays an essential role in various plants. Sung et al. reported that a PPR protein, SLOW GROWTH1 (SLO1), plays a vital role in RNA editing of *NADH dehydrogenase 4* (*nad4*) and *nad9* in the mitochondria of *Arabidopsis*. The *slo1* mutants displayed abnormal phenotypes, such as darker and shrunken seeds, late germination, and delayed development (Sung et al., 2010). Furthermore, another study showed that the mitochondrial editing factor SLO2 was involved in the electron transport chain of mitochondria and hormone and stress responses in *Arabidopsis* (Zhu et al., 2014). *Opaque and growth retardation 1* (*OGR1*) encodes a PPR protein in rice. Kim et al. generated an *ogr1* RNA editing mutant in rice and found that *OGR1* is required for mitochondrial RNA editing in rice and is essential for seed germination and the growth and development of plants (Kim et al., 2009). *Pentatricopeptide repeat 2263* (*PPR2263*) encodes a DYW-subgroup PPR protein responsible for mitochondrial RNA editing in *nad5* and *cytochrome b* (*cob*). Sosso et al. identified the *ppr2263* mutation in maize and found that the mutants exhibited editing defects at the *nad5*-1,550 and *cob*-908 sites. In addition, the authors demonstrated that mitochondrial RNA editing is vital for mitochondrial biogenesis and the growth in maize (Sosso et al., 2012). Another group of scientists showed that the PPR-DYW protein empty pericarp5 is required for mitochondrial RNA editing and seed development in maize (Liu et al., 2013).

Furthermore, abnormal RNA editing could also have adverse effects on the biogenesis of chloroplasts. In *Arabidopsis*, *yellow seedlings 1* (*YS1*) encodes a PPR protein that is localized to chloroplasts and contains a DYW motif. Zhou et al. found that YS1 is essential for the editing of the housekeeping gene *rpoB*. During the early stage, YS1 is required for the differentiation of chloroplasts (Zhou et al., 2009). In rice, *albino seedling lethality 3* (*ASL3*) encodes a chloroplast-localized PPR protein and contains 10 tandem PPR motifs. Lin et al. revealed that interruption of *ASL3* disturbed the transcriptional levels of genes involved in chloroplast development and photosynthesis and caused impaired development of chloroplasts and the growth of seedlings (Lin et al., 2015b).

## Conclusion and Perspectives

RNA editing has aroused great interest because it is a critical supplement to the central dogma. It has been more than 30years since the RNA editing phenomenon was discovered. Various reactions are required for the RNA editing process in plant organelles. To date, some *cis*-elements and different kinds of editing factors have been recognized in plant organelles. These factors are responsible for editing events, including the most common forms of C-to-U and A-to-I conversions. These editing factors are thought to act together with one another to form an RNA editosome. However, there are still many undiscovered *cis*-elements and RNA editing factors. Furthermore, the significance of individual factors and their organization within the editosome is still largely unknown. The mechanism of plant organelle RNA editing needs more exploration in the future.

In recent years, the rapid development of deep sequencing has led to the discovery of numerous RNA editing events in plant organelles. Nevertheless, RNA editing events are not well annotated in some databases, such as DDBJ, GenBank, and ENA. However, some specialized bioinformatics resources have been developed for an appropriate description of plant RNA editing events. Plant RNA editing events are annotated in some databases, including CloroplastDB for plant chloroplast genomes, GOBASE for organellar genomes, PREPACT 3.0, which integrates information on characterized editing factors (Lenz et al., 2018), and REDIdb 3.0, which also shows the changes in amino acids caused by RNA editing events in functional domains and protein secondary structures (Lo Giudice et al., 2018). These databases also facilitate discoveries and functional studies of RNA editing sites in both plant mitochondria and plastids.

## Author Contributions

TQ and DX contributed equally to the design and coordination of the study. WH, GL, and WW collected the data with the help from the WS, YZ, JS, QY, YZ, WF, SP, and ZC wrote the manuscript. All of the authors reviewed and edited the manuscript.

## Funding

This research was supported by Science and Technology Program of Sichuan Province (2020YJ0406), National Natural Science Foundation of China (31900256), Natural Science Foundation of Shandong Province (ZR2020QC026), Modern Agricultural Industry Technical Economic Evaluation System Green Development Position of Henan Province, Soft Science Project of Henan Province (202400410185) and State Key Laboratory of Cotton Biology Open Fund.

## Conflict of Interest

The authors declare that the research was conducted in the absence of any commercial or financial relationships that could be construed as a potential conflict of interest.

## Publisher’s Note

All claims expressed in this article are solely those of the authors and do not necessarily represent those of their affiliated organizations, or those of the publisher, the editors and the reviewers. Any product that may be evaluated in this article, or claim that may be made by its manufacturer, is not guaranteed or endorsed by the publisher.
